# Silicon amendment to rice plants contributes to reduced feeding in a phloem‐sucking insect through modulation of callose deposition

**DOI:** 10.1002/ece3.3653

**Published:** 2017-12-03

**Authors:** Lang Yang, Pei Li, Fei Li, Shahbaz Ali, Xiaoqin Sun, Maolin Hou

**Affiliations:** ^1^ State Key Laboratory for Biology of Plant Diseases and Insect Pests Institute of Plant Protection Chinese Academy of Agricultural Sciences Beijing China; ^2^ Scientific Observing and Experimental Station of Crop Pests in Guilin Ministry of Agriculture Guilin China; ^3^ Southern Regional Collaborative Innovation Center for Grain and Oil Crops in China Changsha China

**Keywords:** β‐1, 3‐glucanase, callose, callose synthase, *Nilaparvata lugens*, phloem feeding, plant defense, rice, silicon amendment

## Abstract

Silicon (Si) uptake by Poaceae plants has beneficial effects on herbivore defense. Increased plant physical barrier and altered herbivorous feeding behaviors are documented to reduce herbivorous arthropod feeding and contribute to enhanced plant defense. Here, we show that Si amendment to rice (*Oryza sativa*) plants contributes to reduced feeding in a phloem feeder, the brown planthopper (*Nilaparvata lugens*, BPH), through modulation of callose deposition. We associated the temporal dynamics of BPH feeding with callose deposition on sieve plates and further with callose synthase and hydrolase gene expression in plants amended with Si. Biological assays revealed that BPH feeding was lower in Si‐amended than in nonamended plants in the early stages post‐BPH infestation. Histological observation showed that BPH infestation triggered fast and strong callose deposition in Si‐amended plants compared with nonamended plants. Analysis using qRT‐PCR revealed that expression of the callose synthase gene *OsGSL1* was up‐regulated more and that the callose hydrolase (β‐1,3‐glucanase) gene *Gns5* was up‐regulated less in Si‐amended than in nonamended plants during the initial stages of BPH infestation. These dynamic expression levels of *OsGSL1* and *Gns5* in response to BPH infestation correspond to callose deposition patterns in Si‐amended versus nonamended plants. It is demonstrated here that BPH infestation triggers differential gene expression associated with callose synthesis and hydrolysis in Si‐amended and nonamended rice plants, which allows callose to be deposited more on sieve tubes and sieve tube occlusions to be maintained more thus contributing to reduced BPH feeding on Si‐amended plants.

## 1 INTRODUCTION

**Figure 1:**
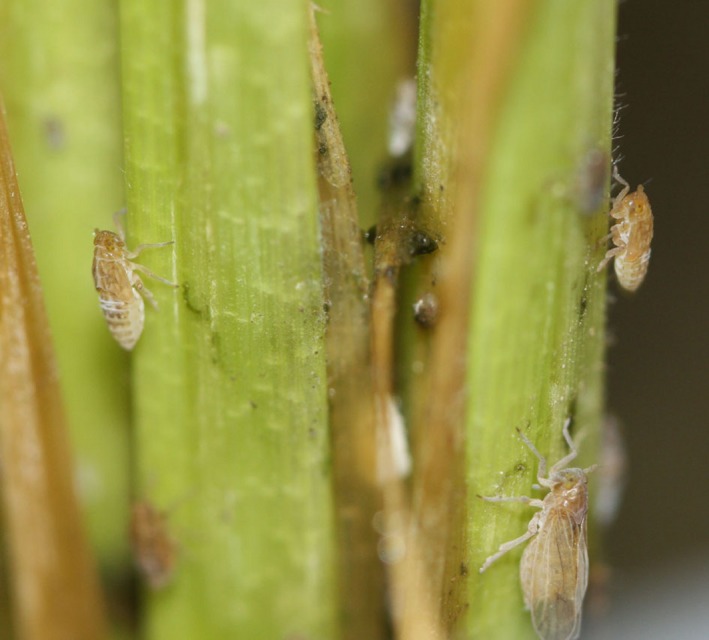
The brown planthopper, *Nilaparvata lugens* Stål, feeding on rice leaf sheath

The brown planthopper (*Nilaparvata lugens* Stål; BPH), a destructive and migratory insect pest, damages rice plants (*Oryza sativa* L.) by ingesting phloem sap via its piercing mouthparts (Bottrell & Schoenly, 2012) (Figure 1). BPH also transmits rice plant viruses (rice grassy stunt virus, rice ragged stunt virus) via salivary excretion into plant phloem (Cabauatan, Cabunagan, & Choi, 2009). Although chemicals can afford substantial control of BPH, it is reported that high insecticide resistance, resurgence of the pest, and high chemical residues in the environment are results of long‐term use and misuse of chemical insecticides (Li et al., 2008). Alternatively, silicon (Si) amendment shows high potential for insect pest management (Hou & Han, 2010; Savant, Snyder, & Datnoff, 1997). In a variety of herbivorous insects, it has been demonstrated that Si amendment to plants can afford substantial plant resistance (Reynolds, Keeping, & Meyer, 2009; Reynolds, Padula, Zeng, & Gurr, 2016). For BPH, reduced performance was recorded on rice plants treated hydroponically with high Si concentrations (He et al., 2015). A recent report determined that Si amendment impaired feeding behaviors and reduced the feeding amount and population growth in BPH (Yang, Han, Li, Wen, & Hou, 2017).

Enhanced plant resistance to herbivores with Si amendment is thought to be the result of a strengthened constitutive defense. The increased rigidity and reduced digestibility of plant tissues due to a physical barrier formed from additional amorphous silica deposition in epidermal cells in Si‐amended plants are the principal components of their heightened constitutive defense (Massey, Ennos, & Hartley, 2006; ;Massey & Hartley, 2009; Han, Lei, Wen, & Hou, 2015). The mechanical barrier afforded by Si amendment can reduce food quality for herbivores and cause wear on the mouthparts, impair feeding behavior and reduce the feeding efficiency and growth rate of herbivores (Calandra, Zub, Szafra_nska, Zalewski, & Merceron, 2016; Han et al., 2015; Hartley, Fitt, McLarnon, & Wade, 2015; Keeping, Kvedaras, & Brutonc, 2009; Massey & Hartley, 2009; Reynolds et al., 2016). However, previous publications about the physical barrier mechanism have been exclusively concerned with vertebrates or chewing insects. Yang, Han, Li, Wen et al. (2017) found that Si amendment to rice plants reduced feeding by BPH and recorded with electrical penetration graph a longer duration of both nonprobing and pathway events in BPH feeding on the plants with high Si addition compared to the control. These results may be directly mediated by the increased abrasiveness and rigidity of plant tissues resulting from intensified silisification of rice leaf sheaths in Si‐added plants (Yang, Han, Li, Wen et al., 2017). The electrical penetration graph also recorded a shorter phloem sap ingestion duration and a lower proportion of individuals that produced sustained phloem sap ingestion on plants with high Si addition than in the control (Yang, Han, Li, Wen et al., 2017), which highlights that the mechanical barrier mechanism only tells part of the story for the reduced BPH feeding.

Si amendment is also reported to be involved in priming the chemical defense in plants (Ghareeb et al., 2011; Han et al., 2016; Yang, Han, Li, Li et al., 2017; Ye et al., 2013) as well as in the augmented release of herbivore‐induced plant volatiles that attract natural enemies of the attacking pests (Kvedaras, An, Choi, & Gurr, 2010; Liu et al., 2017). An important induced plant defense to sucking insects is callose deposition on sieve plates. Callose is a plant polysaccharide formed by hundreds of glucose molecules that are mostly linked by β‐1‐3‐glucosidic bonds (Nedukha, 2015), whose accumulation can be switched on and off by the dynamic roles of callose synthases and hydrolase in response to piercing by sucking insect pests (Hao et al., 2008). When attacked by piercing insects, plants activate callose deposition on sieve plates to occlude the flow of phloem sap to discourage phloem feeding, which is a key resistance mechanism in resistant rice varieties against BPH (Hao et al., 2008). However, it is not clear whether Si is involved in the induction of callose metabolism and deposition and whether this contributes to the reduced feeding by BPH reported by Yang, Han, Li, Wen et al. (2017).

The objectives of this study were to determine whether Si is involved in callose deposition in rice plants attacked by BPH, and if so, whether Si‐mediated callose deposition contributes to reduced feeding by BPH and whether Si is involved in the modulation of callose synthesis. Specifically, with plants not amended with Si and not infested with BPH as the control, BPH feeding was measured dynamically; callose deposition was determined histologically and dynamically and related to the pattern of feeding. Further, the temporal dynamics of callose deposition were linked to the expression of genes encoding callose synthase and hydrolase through quantitative reverse transcriptase PCR (qRT‐PCR). We predict that (1) callose deposition responds positively to Si amendment in BPH‐infested plants, (2) increased callose deposition correlates with reduced feeding by BPH, and (3) Si amendment is involved in the modulation of the gene expression of callose synthase and hydrolase in BPH‐attacked plants. This design has enabled us to disentangle a novel role of Si in enhancing plant resistance to sucking insects, that is, increased callose deposition.

## 2 MATERIALS AND METHODS

### 2.1 Rice plants, Si treatment, and planthoppers

Rice plants of a susceptible variety Taichung Native 1 (TN1) were used to rear the brown planthopper (Yang, Han, Li, Li et al., 2017) and as experimental plants in this study. Briefly, the seedlings were cultured with washed river sand and tap water and were then transplanted to plastic boxes (50 × 40 × 15 cm) at 20 plants per box at 10 days old, where the plants were aquacultured with nutrient solution (Yoshida, Forno, & Cock, 1976), which included NH_4_NO_3_ (114.3 mg/L), NaH_2_PO_4_·2H_2_O (50.4 mg/L), K_2_SO_4_ (89.3 mg/L), CaCl_2_ (110.8 mg/L), MgSO_4_·7H_2_O (405.0 mg/L), MnCl_2_·4H_2_O (1.8 mg/L), Na_2_MoO_4_·2H_2_O (0.126 mg/L), EDTA·Fe·Na (13.25 mg/L), H_3_BO_3_ (1.145 mg/L), ZnSO_4_·7H_2_O (0.044 mg/L), and CuSO_4_·5H_2_O (0.039 mg/L). The solution was prepared using deionized water and brought to a pH of 5.0–6.0 by addition of NaOH or H_2_SO_4_ solutions and was replenished every 5 days from the rice seedling transplanting. Si amendment (+Si) was established at transplanting by adding Na_2_SiO_3_·9H_2_O to the nutrient solution at 112 mg Si/L, and a control without the addition of Na_2_SiO_3_·9H_2_O (–Si) was included. The plants were cultured in a greenhouse (23–32°C, relative humidity (RH) 75%–85%) to exclude rain and naturally occurring pests.

To obtain experimental planthopper populations, adults were periodically transferred from a stock culture maintained on 30 to 45‐days‐old potted TN1 seedlings to insect‐proof cages with 20‐days‐old rice seedlings in climate chambers (RXZ‐260B, Jiangnan Instrument Plant, Ningbo, China) at 26 ± 1°C, RH 85% ± 5%, and 14L:10D for oviposition. After 24 hr, the seedlings were removed from the oviposition cages and maintained in new cages with rice seedlings until the nymphs therein reached the 5th stadium when they were transferred to glass tubes (2.5 × 15 cm) with aquacultured rice seedlings. Newly emerged macropterous female adults were used in the experiments. The plants used for insect rearing were not amended with Si.

### 2.2 Quantification of BPH feeding

The amount of feeding was recorded as a measure of honeydew excretion using a parafilm sachet method (Pathak, Saxena, & Henrichs, 1982). One newly emerged macropterous female was confined in a parafilm sachet (4 × 4 cm) with the opening attached to the stem of a 30‐days‐old +Si or –Si rice seedling, where the insect can feed freely on the rice sheath through the opening of the sachet (Pathak et al., 1982). At 24, 48, 72, or 96 hr postinfestation (hpi), the insect was removed and the sachet was weighed immediately. The net weight of the honeydew was obtained by deduction of the blank sachet weight from the final sachet weight. The experiment was performed under laboratory conditions of 25–28°C and RH 85% ± 5%. Each insect served as a replicate. Twenty insects were tested for each +Si and –Si plant at each of 24, 48, 72, and 96 hpi.

### 2.3 Histological observation of callose deposition

This observation was conducted to determine whether callose deposition would respond positively to Si amendment in BPH‐infested rice plants and its relationship with the BPH feeding amount. A +Si or –Si rice seedling was exposed to BPH by the parafilm sachet method (Pathak et al., 1982). In brief, a sachet (4 × 4 cm) was fastened to the middle of the stem of a 30‐days‐old seedling in the climatic chamber, into which five newly emerged female adults were transferred. At 24, 48, 72, and 96 hr postinfestation (hpi), that is, the total time that BPH was allowed to feed on the plants, the sachet was removed, and the segment of the leaf sheath damaged by BPH was collected using a blade. Plants not exposed to BPH damage were also sampled in the same way.

Cross‐paraffin sections of the leaf sheath samples (0.2 cm long) were obtained using a microtome (Meditome M530, German). Briefly, a leaf sheath sample was immersed into FAE (formaldehyde:acetic acid:70% ethanol = 5:5:90 (v:v:v)) fixing solution for 2 days and then dehydrated for 2–3 hr using dimethylbenzene. Thereafter, the leaf sheath was wrapped with paraffin and sliced into cross‐sections of 10 μm thickness. More than 40 cross‐sections were obtained from a leaf sheath per plant. After the sections were stained for 5 min in 0.1% (w/v) aniline blue, they were flushed with tap water and then, with water on the surface removed using absorbent paper, air dried. A dried section was loaded onto a glass slide and observed under UV light using a fluorescence microscope (Olympus BX63, Japan) to record the number of sieve plats with callose deposition in vascular bundles according to Hao et al. (2008). Sieve plates with bright blue fluorescence were recorded as callosic plates (McNairn & Currier, 1967). The number of callosic sieve plates was recorded from 40 cross‐sections per plant, and 10 plants were observed for each treatment. Photographs were obtained using a digital camera (Olympus DP73, Japan).

### 2.4 Gene expression of callose synthase and hydrolase

Callose deposition was previously shown to be correlated with the mRNA levels of the genes encoding callose synthase and the callose hydrolase β‐1,3‐glucanase (Hao et al., 2008). The glucan synthase‐like gene (*OsGSL1*) is the principal callose synthase‐encoding gene, and *Gns5* is the main gene encoding β‐1,3‐glucanase (Hao et al., 2008). To account for the dynamic expression of callose deposition, we quantified the relative expression levels of *OsGSL1* (Accession number AP001389) and *Gns5* (Accession number U72251) by qRT‐PCR (Hao et al., 2008).

Leaf sheath samples were harvested in the same way as for histological observation, but the time points were 0, 3, 6, 12, 24, 48, 72, or 96 hpi, that is, the total time that BPH was allowed to feed on the plants. The samples were stored at −80°C immediately after collection. Total RNA was extracted from the four leaf sheaths of two rice plants collected at a certain time post‐BPH infestation from each treatment using RNAiso plus Reagent (TaKaRa, Dalian, China) following the manufacturer's instructions. For each total RNA sample, 1.2 μg of RNA was reverse‐transcribed to cDNA by the Fast Quant RT Kit (Tiangen, Beijing, China). Two references genes *Actin1* (Accession number AB047313) and *UBQ5* (Accession number AK061988) were used for normalization. The primers synthesized according to Hao et al. (2008) were as follows: *OSGSL1*‐F: 5′‐TGCCCATTGTTCTTTTCA‐3′; *OSGSL1*‐R: 5′‐TCCTGATTTGCCTTGTTTCC‐3; *Gns5*‐F: 5′‐ATTGGTCCTTGGATTGCG‐3′; and *Gns5*‐R: 5′‐CGATGCCGTTGGACTTGTA‐3′. The reference genes were synthesized according to Du et al. (2009) and Li, Li, Zhou, and Lou (2013), as follows: *Actin1*‐F: 5′‐CAGCACATTCCAGCAGAT‐3′; Actin1‐R: 5′‐GGCTTAGCATTCTTGGGT‐3′; *UBQ5*‐F: 5′‐AACCACTTCGACCGCCACT‐3′; and *UBQ5*‐R: 5′‐GTTCGATTTCCTCCTCCTTCC‐3′. The specificity and efficiency of each primer were verified by analyzing standard curves of a tenfold cDNA dilution series. Every attempt was made to adhere to minimum information for quantitative real‐time PCR experiments guidelines to ensure proper and accurate reporting of qRT‐PCR data (Bustin et al., 2009).

The qPCR was carried out on the ABI 7500 Real‐Time PCR System (Applied Biosystems, Carlsbad, CA, USA) in a 20 μl mixture containing 10 μl of Bester^®^ SybrGreen qPCR MasterMix (DBI^®^ Bioscience, Germany), 4 μl (20 ng) of sample cDNA, 0.4 μl of each primer, 0.04 μl 50× ROX Reference Dye, and 5.16 μl of RNase‐free H_2_O. The qPCR cycling conditions were 95°C for 5 min, followed by 40 cycles of 95°C for 10 s and 60°C for 31 s, melt curves stages at 95°C for 15 s, 60°C for 1 min, and 95°C for 15 s. Negative controls without template were included in each experiment. The qPCR was performed in triplicate, and the results were averaged for each of three biological replicates for a certain time post‐BPH infestation per treatment. The relative expression levels of *GNS5* and *OSGSL1* were calculated with the 2^−ΔΔCt^ method (Livak & Schmittgen, 2001). The averages of the Ct values of both reference genes were used to calculate ΔΔCts of the target genes with the equation: ΔΔCt = (Average Ct_target_ – Average Ct_refference_) _sample i_ – (Average Ct_target_ – Average Ct_refference_) _sample CK_, where sample CK is the sample at 0 hpi (plants not infested by BPH).

### 2.5 Statistical analysis

The data of BPH feeding, callose deposition, and gene expression were subjected to an analysis of variance (ANOVA) for the effects of Si amendment, BPH infestation duration, and their interactions. Tukey HSD test was used as post hoc test to separate the differences between treatments. Additionally, the difference between +Si and –Si plants at a certain hpi was compared by independent samples *t* test (*p *=* *.05). All statistical analysis was performed using SPSS 16.0 (SPSS Inc, USA).

## 3 RESULTS

### 3.1 BPH feeding

**Figure 2:**
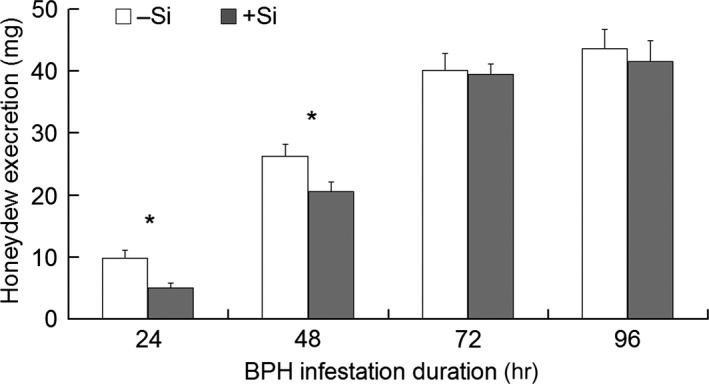
Honeydew excretion (indicative of feeding amount) of BPH macropterous females feeding on rice leaf sheaths in response to Si amendment. +Si = Si amendment to rice plants at 112 mg Si/kg nutrient solution; –Si = no Si amendment. Error bars represent 1 × *SE*. *n *=* *20 (biological replicates). An asterisk (*) indicates significant difference between +Si and –Si plants at a certain time post‐BPH infestation at *p < *.05 via independent samples *T* test

Honeydew excretion was significantly influenced by Si treatment (*F*_1,160 _= 4.50, *p *=* *.036), feeding duration (*F*_3,160 _= 108.94, *p *<* *.001), and their interaction (*F*_1,160 _= 108.94, *p *<* *.001). Honeydew excretion was higher at 72 and 96 hpi than at 24 and 48 hpi and higher at 48 hpi than at 24 hpi (Tukey HSD tests, *p *<* *.015), whether the plants were Si‐amended or not. Between the Si treatments, higher honeydew excretion was observed on +Si plants than on –Si plants at 24 (*t*_38 _= 3.06, *p *=* *.004) and 48 (*t*_38 _= 2.33, *p *=* *.025) hpi (Figure 2).

### 3.2 Callose deposition

**Figure 3:**
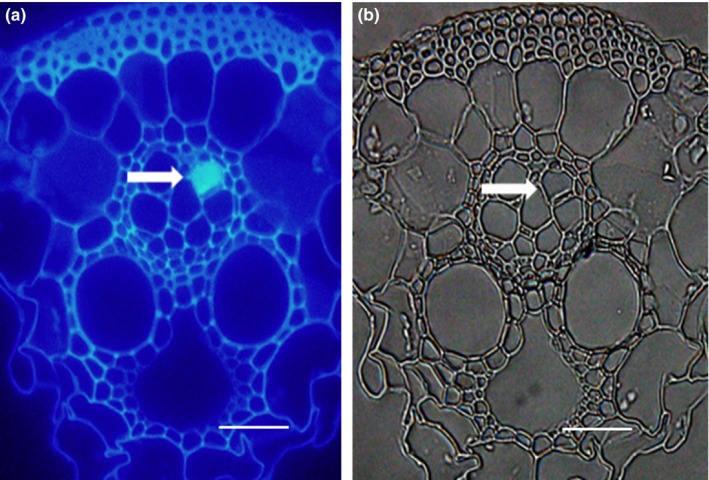
Callose deposition in rice leaf sheath tissues obtained with fluorescence microscope at 40 × . White arrows show induced callose (with bright blue fluorescence) deposited on the sieve plates in the Si‐amended and BPH‐infested rice plants. (a) Obtained under ultraviolet. (b) Light microphotographs of a. Scale bar = 50 μm

**Figure 4:**
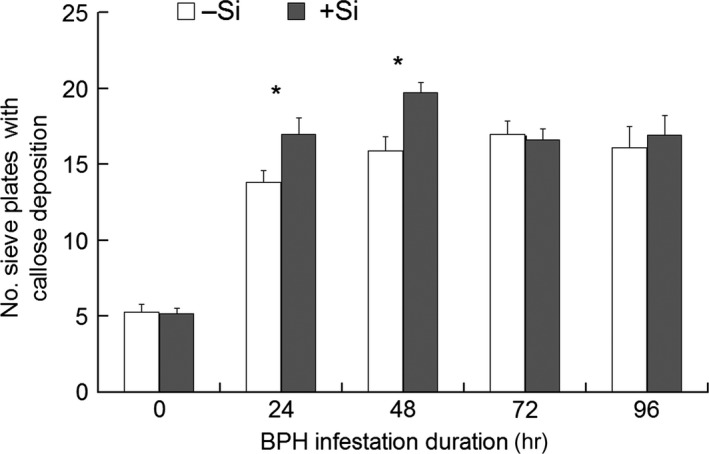
Callose deposition in rice leaf sheaths in response to Si amendment and BPH infestation. +Si = Si amendment to rice plants at 112 mg Si/kg nutrient solution, –Si = no Si amendment. The histogram bars represent average number of sieve plates with callose deposition observed in the 40 cross‐sections obtained per leaf sheath from 10 different plants per treatment. Error bars represent 1 × *SE*. An asterisk (*) indicates significant difference between +Si and –Si plants at a certain time post‐BPH infestation at *p < *.05 via independent samples *T* test

The sieve plates deposited with callose were obviously thickened and emitted strong fluorescence (Figure 3). It was found that both the Si treatment (*F*_1,100 _= 6.27, *p *=* *.014) and feeding duration (*F*_4,100 _= 62.65, *p *<* *.001) significantly influenced the number of sieve plates with callose deposition (Figure 4). The number of callosic sieve plates underwent a significant increase from 5.3 at 0 hpi (i.e., not infested, the data of +Si and –Si plants were pooled together) to 15.3 or more after the plants were infested by BPH (+Si and –Si pooled) in 40 sections (Tukey HSD test, *p *<* *.001). With prolonged BPH feeding, callose deposition reached its peak at 48 hpi in +Si plants and at 72 hpi in –Si plants and then decreased in both +Si and –Si plants (Figure 4). Higher callose deposition was observed in +Si plants than in –Si plants at 24 hpi (*t*_18 _= 2.40, *p *=* *.027) and 48 hpi (*t*_18 _= 3.36, *p *=* *.003).

### 3.3 Expression of callose synthase and hydrolase

**Figure 5:**
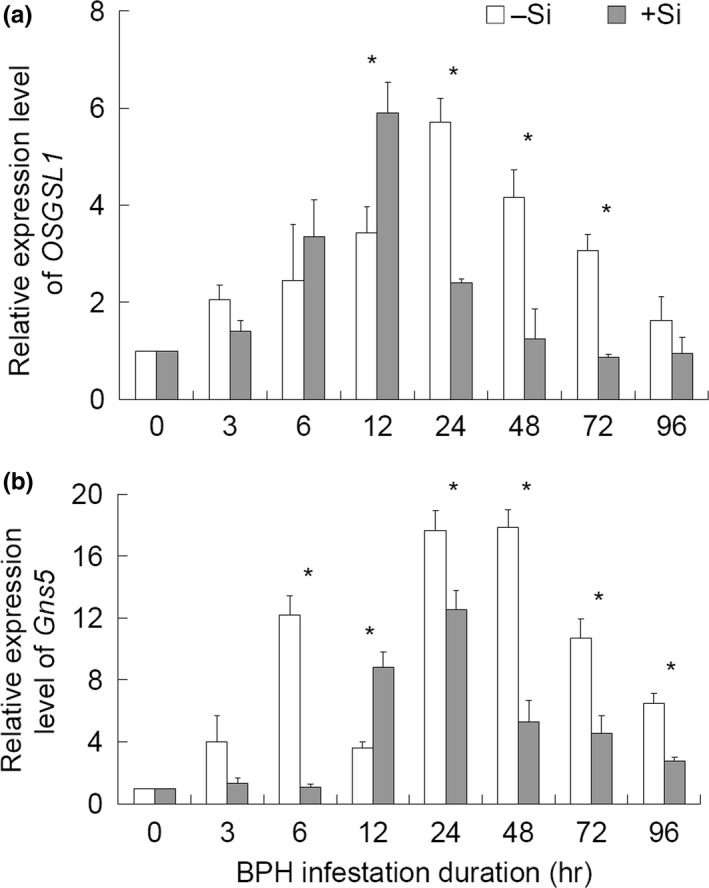
Relative expression levels of the genes responsible for callose synthesis and hydrolysis in the rice sheath in response to Si amendment and BPH infestation. (a) Callose synthase gene, *OSGSL1*. (b) β‐1,3‐glucanase gene, *GNS5*. +Si = Si amendment to rice plants at 112 mg Si/kg nutrient solution, –Si = no Si amendment. Error bars represent 1 × *SE*. *n *=* *3 (biological replicates). Total RNA was extracted from rice leaf sheaths obtained at various hours post‐BPH infestation; gene expression was quantified relative to the value for 0 hr samples (BPH‐free plants). Each RNA sample was extracted from four fresh leaf sheaths of two rice plants from a treatment. Genes *Actin1* and *UBQ5* were used as reference control. An asterisk (*) indicates significant difference between +Si and –Si plants at a certain time post‐BPH infestation at *p < *.05 via independent samples *T* test

The expression of *OsGSL1*, the callose synthase‐encoding gene, and *Gns5*, the main gene encoding β‐1,3‐glucanase, was measured using qRT‐PCR. *OsGSL1* was obviously up‐regulated by BPH infestation at 3 hpi (ca. 2‐fold of uninfested plants), reaching the peak at 12 hpi in +Si plants (5.9‐fold of uninfested plants) and at 24 hpi in –Si plants (5.7‐fold of uninfested plants) in a hump shape (Figure 5a). The expression levels decreased abruptly in +Si plants at 24 hpi and remained low during 48–96 hpi (at approximately the same level of uninfested plants), while in –Si plants, they decreased gradually from 48 hpi. Significant influence of Si treatment (*F*_1,48 _= 9.84, *p *=* *.004), feeding duration (*F*_7,48 _= 10.16, *p *<* *.001), and their interaction (*F*_7,48 _= 5.76, *p *<* *.001) on *OsGSL1* expression was found. Between the Si treatments, a significantly higher (*t*_4 _= 2.94, *p *=* *.042) expression of *OsGSL1* was found in Si‐amended plants than in nonamended plants at 12 hpi while at 24, 48, and 72 hpi, it was higher (*t*_4 _≥ 3.52, *p *≤* *.024) in –Si plants than in +Si plants (Figure 5a). *Gns5* expression was also clearly up‐regulated by BPH infestation at 3 and 6 hpi in –Si plants (4‐ to 12‐fold of the uninfested plants) while in +Si plants, it remained at a similar level as the uninfested plants until 12 hpi, when the expression level increased abruptly by over 6.7‐fold compared to those at 0, 3 or 6 hpi (Figure 5b). Significant influence of Si treatment (*F*_1,48 _= 244.95, *p *<* *.001), feeding duration (*F*_7,48 _= 126.56, *p *<* *.001), and their interaction (*F*_7,48 _= 49.34, *p *<* *.001) on *Gns5* expression was also recorded. Between the Si treatments, the *Gns5* expression in –Si plants was higher than that in +Si plants at 6, 24, 48, 72, and 96 hpi (*t*_4 _≥ 2.82, *p *≤* *.048) and was lower than that in +Si plants only at 12 hpi (*t*_4 _= 4.86, *p *=* *.008) (Figure 5b).

## 4 DISCUSSION

Our present study confirmed the results of Yang, Han, Li, Wen et al. (2017) that Si‐amended rice plants reduced honeydew excretion and therefore feeding of BPH. Furthermore, we were able to demonstrate that callose deposition responded positively to BPH infestation in both +Si and –Si plants, as indicated by more callosic sieve plates in the BPH‐infested than in the uninfested plants (Figure 4). More interestingly, +Si plants harbored more callosic sieve plates than –Si plants at 24 and 48 hpi (Figure 4), which corresponds to the significantly reduced feeding by BPH on +Si than on –Si plants at 24 and 48 hpi (Figure 2). These data indicate an apparent connection between callose depositions and reduced BPH feeding. It may be reasoned that the increased callose deposition in +Si plants relative to that in –Si plants functions to occlude phloem sap flow and shortens/reduces phloem sap ingestion by BPH, which is similar to the way that callose functions in a resistant rice variety against BPH (Hao et al., 2008). Although callose functions to enhance plant resistance to sucking insects by the occlusion of phloem sap flow, it does not belong to the paradigm of mechanical barrier mechanism that is directly caused by Si accumulation in epidemic cells. In nature, it is an induced defense, as callose deposition is triggered by piercing wounding from sucking insect pests. Therefore, these results have furthered our understanding of silicon's role in plants’ induced defense against herbivores.

Callose deposition is a process that is coordinated through the expression of the genes encoding callose synthase (*OsGSL1*) and hydrolase (*Gns5*) (Hao et al., 2008). Our data indicate that BPH infestation induces high expression of the callose synthase‐encoding gene (*OsGSL1*) and low expression of the gene encoding callose hydrolase (*Gns5*) in +Si plants relative to –Si plants (Figure 5). Confirming the dynamic expression levels of both *OsGSL1* and *Gns5*, callose deposition is higher in +Si than in –Si plants at 24 and 48 hpi (Figure 4), which may allow the sieve tube occlusions to be maintained more in +Si than in –Si plants for a short duration post‐BPH infestation. This is probably one of the reasons for the shorter phloem sap ingestion duration and lower proportion of individuals with sustained phloem sap ingestion (Yang, Han, Li, Wen et al., 2017) as well as the reduced feeding in this study for BPH in Si‐amended rice plants. However, starting from 24 and 48 hpi, the expression of *OSGS1* and *Gns5* tended to decrease in both +Si and –Si plants and the number of callosic sieve plates decreased beginning at 72 hpi. It can be reasoned that the expression of *OSGS1* and *Gns5* and accumulation of callose is a dynamic stress response of the plants to herbivorous infestation and is demanding on the stressed plants. The increased callose deposition will hamper plant physiology and development if it is maintained for a longer duration. From an evolutionary point of view, it is of benefit for the stressed plants to be selected for a short‐lived stress response. This short‐lived stress response can “drive” the herbivores away and/or reduce herbivores’ performance, thus exposing them more to natural control factors, such as natural enemies and adverse climatic conditions. Our results show that Si amendment helps the plants achieve this “goal.”

In summary, our results show that Si amendment adds to fast and strong callose deposition in plants infested by BPH, which is connected with the lower BPH feeding amount on Si‐supplemented plants in the early stages post‐BPH infestation. Our results further reveal that Si amendment is involved in modulation of the gene expression of callose synthase and hydrolase triggered by BPH infestation and that high expression of the synthase gene *OsGSL1* and low expression of the hydrolase gene *Gns5* are recorded in Si‐amended plants during the initial stages of BPH infestation. Taken together, our findings demonstrate that Si amendment to rice plants contributes to reducing BPH feeding through modulation of callose deposition, a novel role of silicon in enhancing plant resistance to sucking insect pests.

## CONFLICT OF INTEREST

The authors have no conflict of interest to declare.

## AUTHOR'S CONTRIBUTIONS

MH and LY conceived of the ideas and designed the methodology; LY, PL, FL, SA, and XS collected the data; LY and MH analyzed the data; and LY and MH led the writing of the manuscript. All authors contributed critically to the drafts and gave final approval for publication.

## DATA ACCESSIBILITY

All data used in this manuscript are present in the manuscript, and there is no data archiving.
